# Case report: Laparoscopic nissen-sleeve gastrectomy in a young adult with incidental finding of Morgagni-Larrey hernia

**DOI:** 10.3389/fsurg.2023.1227567

**Published:** 2023-07-21

**Authors:** Rossella Palma, Francesco Angrisani, Antonella Santonicola, Paola Iovino, Vittorio Maria Ormando, Roberta Maselli, Luigi Angrisani

**Affiliations:** ^1^ Department of Surgical Sciences, “Sapienza” University of Rome, Rome, Italy; ^2^ Humanitas University, Milan, Italy; ^3^ Department of Medicine, Surgery and Dentistry “Scuola Medica Salernitana”, University of Salerno, Salerno, Italy; ^4^ Gastroenterology and Endoscopy Unit, AORN San Giuseppe Moscati, Avellino, Italy; ^5^ Endoscopy Unit, Humanitas Clinical and Research Center IRCCS, Rozzano, Italy; ^6^ Department of Biomedical Sciences, Humanitas University, Milan, Italy; ^7^ Department of Public Health, “Federico II” University of Naples, Naples, Italy

**Keywords:** obesity, diaphragmatic hernia, Morgagni-Larrey hernia, hiatal hernia, sleeve gastrectomy

## Abstract

Laparoscopic sleeve Gastrectomy (LSG) is the most performed bariatric procedure worldwide but it is associated with an increased incidence of de-novo or recurrent GERD. Recently a new technique consisting in LSG with associated fundoplication has been described. Morgagni-Larrey hernia is very rare and there is a lack of evidences on its correct surgical treatment. There are only rare cases of a MLH incidental diagnosis in patients submitted to bariatric surgery. We present our experience of Morgagni-Larrey Hernia (MLH) incidentally found intraoperatively in a patient with Gastroesophageal Reflux Disease (GERD) with Hiatal Hernia (HH) undergoing a bariatric surgical procedure.

## Introduction

Laparoscopic sleeve Gastrectomy (LSG) is the most performed bariatric procedure worldwide (1). It is known that LSG is associated with an increased incidence of de-novo or recurrent GERD (2) and Barrett’s Esophagus (BE) (3). Roux-en-Y Gastric Bypass (RYGB) is considered the first choice in patients affected by GERD and Hiatal Hernia. Several studies investigated the effectiveness of LSG with associated HH repair (HHR), although the long-term results are contradictory and HH and GERD symptoms recurrence have been described (4, 5).

Recently a new technique consisting in LSG with associated fundoplication has been described (6). To the best of our knowledge, there are only few anecdotal cases of intraoperative finding and concomitant repair of a Morgagni-Larrey diaphragmatic hernia during a bariatric procedure (7, 8).

## Case description

In our experience, a 18-year-old male patient with grade III obesity (BMI = 40.76 Kg/m^2^) and GERD symptoms was referred to our center to receive bariatric surgery. The patient previously placed an intragastric balloon with an initial weight loss of 30 kg, however by two years more than 80% of lost weight was regained. The preoperative upper gastrointestinal endoscopy (UGIE) showed a three centimeters grade 4 HH according to Hill Classification. HH was confirmed on X-ray esophagus and no other defects were preoperatively diagnosed. The patient presented typical GERD symptoms that severely impacted his quality of life. We decided to perform a LSG with associated Nissen fundoplication and HHR.

**Figure 1:**
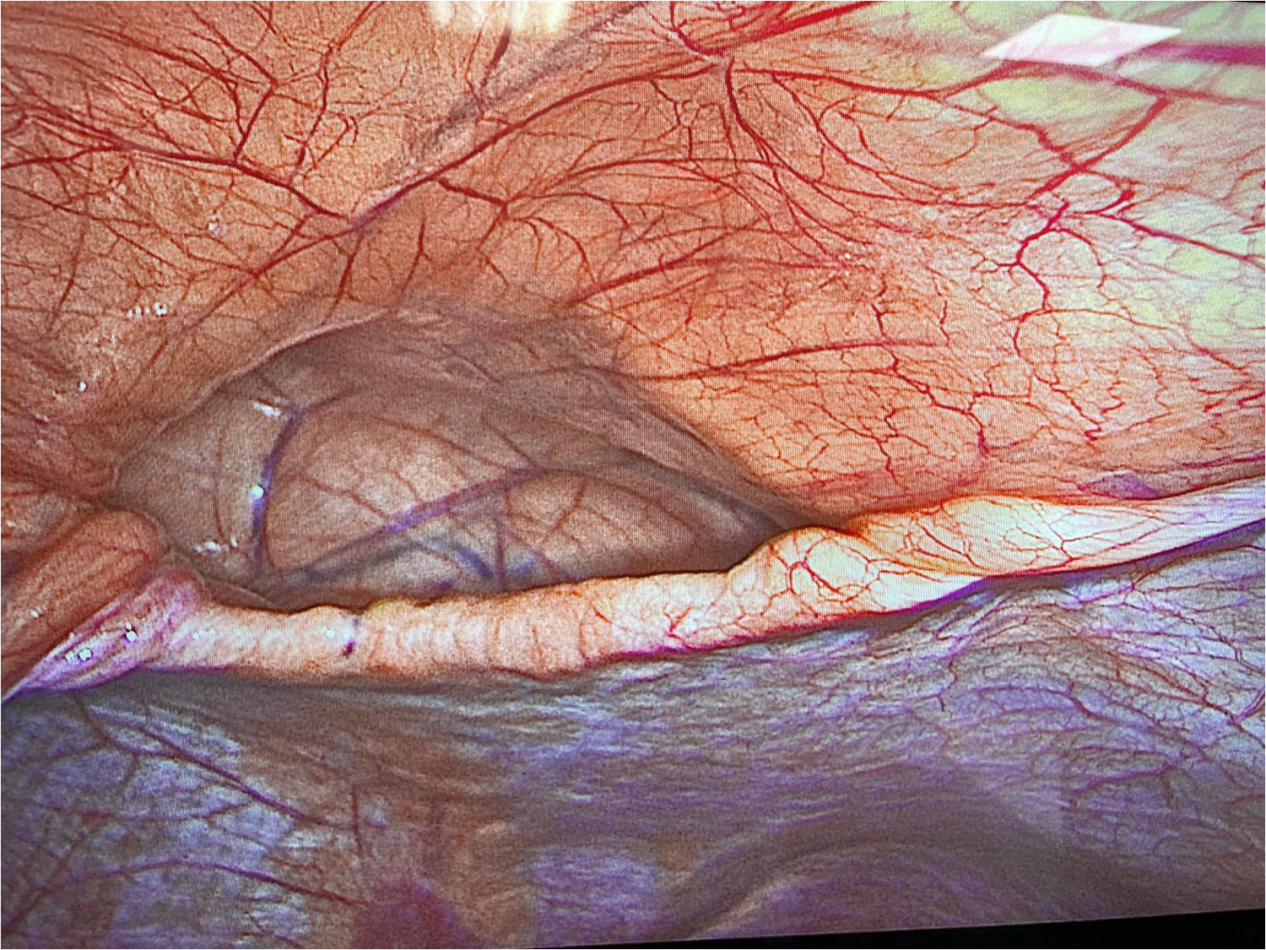
Intraoperative finding of a Morgagni-Larrey hernia.

**Figure 2:**
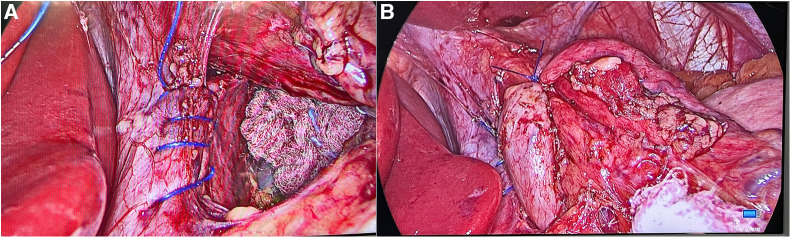
Nissen fundoplication with associated HHR. (**A**) Posterior cruroplasty by running barbed suture technique; (**B**) 360° antireflux valve.

During surgery a defect in the anterior, parasternal portion of the diaphragm was immediately recognized (Figure 1). The diaphragmatic hernia sac appeared free of content and the choice was to perform the planned bariatric procedure without repairing the defect (Figure 2). No perioperative complications were recorded and the patient was discharged 2 days later. At the 6 months follow up visit, the BMI was 31.14 kg/m^2^ with a TWL of 23.6%. Furthermore, the patient didn’t report GERD symptoms, dyspepsia, dyspnea and abdominal or chest pain.

## Discussion

Morgagni-Larrey hernia (MLH) represents the most common congenital defect in the anterior, parasternal portion of the diaphragm. This hernia is very rare with a reported incidence ranging from 3% to 5% of all diaphragmatic hernias and it is most frequently diagnosed in infants and children. However, the hernia can remain undiagnosed until adulthood, due to a vague clinical presentation (9). The patients’ symptoms are generally unspecific and range from dyspnea to abdominal or chest pain and constipation. However, these are mostly dependent on hernia sac content with small or large intestine, as well as on the hernia size with compressive symptoms such as dyspnea, fatigue or exercise intolerance.

The exact prevalence of MLH in adult patients remains unclear and the knowledge about diagnostic methods, imaging techniques and treatment modalities is only based on single surgeon’s experiences and anecdotal reports. In our clinical practice, over the last thirty years, only one case of pre-operative finding of Morgagni-Larrey hernia was found in a lean patient with epigastric pain and subocclusive symptoms (10) which underwent hernia reduction and direct suture repair. No other similar cases have been observed in our bariatric experience.

Although several studies report the successful repair of MLH in infants. According to a recent review with meta-analysis the recurrence and complication rates are comparable between mini invasive approaches and open repairs in patients with mean age of 17-months. Anyway the use of patch appeared to confer additional benefit in reducing recurrence (11). Recently has been also described the laparoscopy-assisted transabdominal repair of MLH using loop suture, with successful results. Leaving the hernia sac apparently does not increase the recurrence rate (12).

The indication and operative strategies of Morgagni-Larrey hernia in adults remain controversial. In a large retrospective single-center study on adult patients with MLH, surgery was performed safely with mesh reinforcement after primary closure (13).

The possible described surgical treatment options of an extra-hiatal diaphragmatic hernia were:

- primary suture
- primary suture with mesh reinforcement
- mesh interposition without primary closure

In conclusion the evidences about the diagnostic pathway and surgical options in adult patients are very low, especially when incidentally discovered during a bariatric procedure. The symptoms are generally unspecific and could be overlapping to other clinical conditions, especially in case of a concomitant hiatal hernia in an obese patient.

Although MLH could be safely repaired with a laparoscopic access taking into account the surgeon experience and patient’s symptoms, in our case the extra-hiatal hernia was incidentally diagnosed. The evidence of an empty sac, the lack of specific symptoms associated with the MLH, and the absence of a specific surgical informed consent (being found incidentally) have led the surgeon to prefer a conservative approach, leaving intact the MLH, to give priority to the treatment of obesity and GERD.

## Data Availability

The data that support the finding of this study are available from the corresponding author upon reasonable request.
